# A new mini-open technique of arthroscopically assisted Latarjet

**DOI:** 10.1186/s12891-020-03307-0

**Published:** 2020-05-07

**Authors:** Ettore Taverna, Umile Giuseppe Longo, Vincenzo Guarrella, Guido Garavaglia, Carlo Perfetti, Luca Maria Sconfienza, Laura Broffoni, Vincenzo Denaro

**Affiliations:** 1 IRCCS Istituto Ortopedico Galeazzi, Milan, Italy; 2 Department of Orthopaedic and Trauma Surgery, Campus Bio-Medico University, Via Alvaro del Portillo, 200, 00128 Trigoria, Rome Italy; 3 Servizio di Chirurgia dell’Arto Superiore, OBV, Mendrisio, Switzerland

**Keywords:** Arthroscopic Latarjet, Mini-open, Arthroscopy, Latarjet, Complications, Endobutton

## Abstract

**Background:**

The aim is to describe a new arthroscopically assisted Latarjet technique.

**Methods:**

We evaluated the clinical and radiological findings of 60 patients with chronic recurrent anterior gleno-humeral instability who underwent, between September 2013 and November 2014, an arthroscopically-assisted Latarjet procedure with double round endobutton fixation. Inclusion criteria were: chronic anterior recurrent instability, Instability Severity Index Score (ISIS) greater than three points, a glenoid bone defect > 15% or a Hill Sachs lesion with concomitant glenoid bone defect > 10%. During surgery the joint capsule and the anterior glenoid labrum were detached. Two drill tunnels perpendicular to the neck of the glenoid were made through a guide. An accessible pilot hole through the glenoid was created to allows the passage of guidewires for coracoid guidance and final fixation onto the anterior glenoid. Through a restricted deltopectoral access a coracoid osteotomy was made. Finally, the graft was prepared, inserted and secured using half-stitches.

**Results:**

The mean follow-up was 32.5 months (range 24–32 months). At a mean follow-up, 56 of the 60 subjects claimed a stable shoulder without postoperative complaints, two (3.3%) had an anterior dislocation after new traumatic injury, and two (3.3%) complained of subjective instability. At the latest follow-up, four subjects complained of painful recurrent anterior instability during abduction-external rotation with apprehension. At 1 year, the graft had migrated in one patient (1.7%) and judged not healed and high positioned in another patient (1.7%). Moreover, a glenoid bony gain of 26.3% was recorded. At the latest follow-up, three patients had grade 1 according to Samilson and Prieto classification asymptomatic degenerative changes. Nerve injuries and infections were not detected. None of the 60 patients underwent revision surgery. Healing rate of the graft was 96.7%.

**Conclusions:**

This technique of arthroscopically assisted Latarjet combines mini-open and arthroscopic approach for improving the precision of the bony tunnels in the glenoid and coracoid placement, minimizing any potential risk of neurologic complications. It can be an option in subjects with anterior gleno-humeral instability and glenoid bone defect. Further studies should be performed to confirm our preliminary results.

**Trial registration:**

Trial registration number 61/int/2017

Name of registry: ORS

Date of registration 11.5.2017

Date of enrolment of the first participant to the trial: September 2013 ‘retrospectively registered’

**Level of evidence:**

IV

## Background

The Latarjet procedure is a popular procedure to manage recurrent shoulder instability [1].

Recently, the arthroscopic technique has also been applied to the Latarjet procedure [2–4]. Concerns arise from the technical challenges of arthroscopy, which may cause an elevated incidence of complications [5, 6].

In fact, despite the excellent and reproducible results of the Latarjet procedure in terms of stability, the incidence of complications (graft malpositioning, nonunion, graft migration, graft fracture. Hardware problems, and nerve damage) is around 15% in open Latarjet [7, 8]. Moreover, the arthroscopic procedure requires more time and higher costs [8–10] .

Coracoid graft malpositioning is one of the most common complications [11]. When grafts are placed too medially, redislocation may occur [12]. On the other hand, when grafts are placed too laterally, degenerative changes may occur [11, 12].

If the screw direction is not correct, the screw may be too prominent laterally and it may impact with the head of the humerus and the scapular spine damaging the joint and potentially the suprascapular nerve [11, 13, 14]. Neurologic complications have been described with an incidence of 3.1–10% [15, 16].

The arthroscopic technique allows better positioning of the tunnels, whereas the open procedures have the advantages of a better preparation of the graft.

To overcome these difficulties, we propose a combined mini-open and arthroscopic approach for improving the precision of the osseous tunnels in the glenoid and the coracoid placement, minimizing any potential risk of neurologic complications. The present technique takes inspiration from Boileau’s procedure for Latarjet and Taverna’s method for bone-block performed through a mini-open approach [7, 17].

The parallel drilling of the two tunnels perpendicular to the glenoid neck can be obtained through an arthroscopic guide that aims to decrease the risk of non-union and graft resorption [17, 18].

The mini-open preparation of the graft allows safe preparation, avoiding the risk of nerve injuries which may occur during the arthroscopic technique, because of the use of portals medial to the coracoid [5, 6, 8, 19].

This study aims to describe an arthroscopically assisted Latarjet, which combines a mini-open and arthroscopic approach for improving the precision of the osseous tunnels in the glenoid and the coracoid placement, minimizing any potential risk of neurologic complications for subjects with glenoid bone deficiency and anterior instability.

## Methods

The study is a retrospective consecutive case series.

The local Ethics committee of Ospedale San Raffaele of Milan approved the present study. All participants were enrolled at “Istituto Ortopedico Galeazzi” of Milan.

### Patients

Between September 2013 and November 2014, 60 patients undergoing an arthroscopically-assisted Latarjet procedure with double round endobutton fixation were enrolled.

Inclusion criteria were: chronic anterior recurrent instability (more than five dislocating episodes and first episode having occurred more than 3 years before), Instability Severity Index Score (ISIS) [20] greater than three points, a glenoid bone defect > 15% measured through preoperative CT scan [21] and confirmed arthroscopically, or a Hill Sachs lesion with concomitant glenoid bone defect > 10% measured through preoperative CT scan and confirmed arthroscopically [22, 23]. Exclusion criteria were: first-time dislocation, no clear dislocation episodes, posterior bone loss [24], voluntary or multidirectional instability [25], glenoid bone deficit of less than 10%, glenoid fractures (apart from bone loss), isolated Hill-Sachs lesion without glenoid bony defect [26] and patients underwent to previously failed anterior stability repair.

The “best-fit circle” technique on preoperative Computer Tomography [21] with Osirix software (Pixmeo, Geneva, Switzerland) was used to measure the glenoid bone loss.

### Surgical technique

Beach chair position without any traction to allow intra-operative mobilization is used. The surgeon starts performing the joint inspection; then the joint capsule and the anterior glenoid labrum are detached. At this point, the surgeon frees the capsulo-labral complex, visualizing the subscapularis tendon. The anterior osseous defect is polished through the motor-powered burr.

**Fig. 1:**
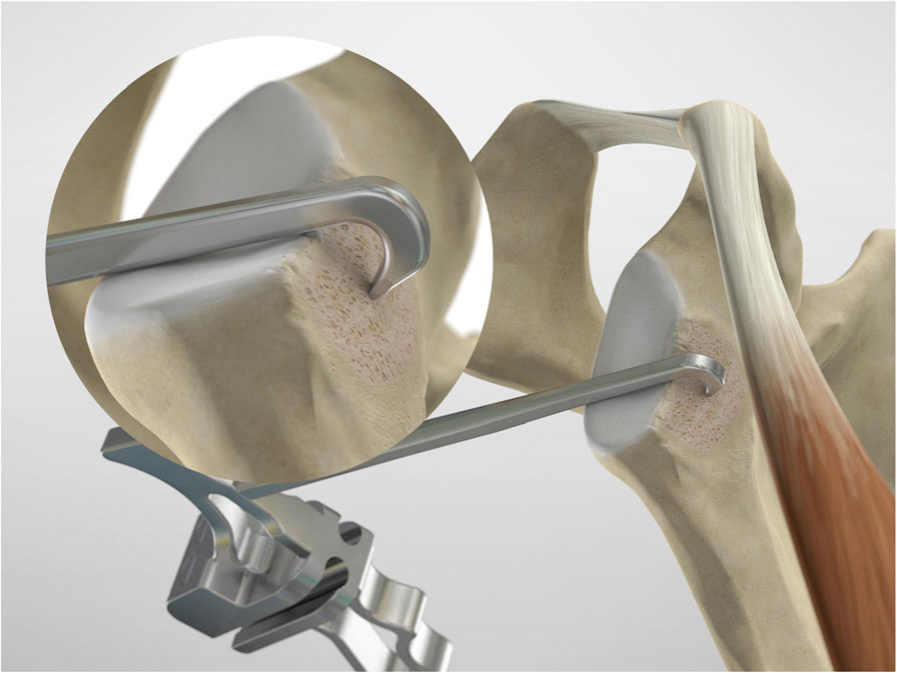
The drill guide (Smith & Nephew Inc., USA) is used to ensure the correct position of two drill tunnels, which should be parallel to each other and perpendicular to the glenoid neck

**Fig. 2:**
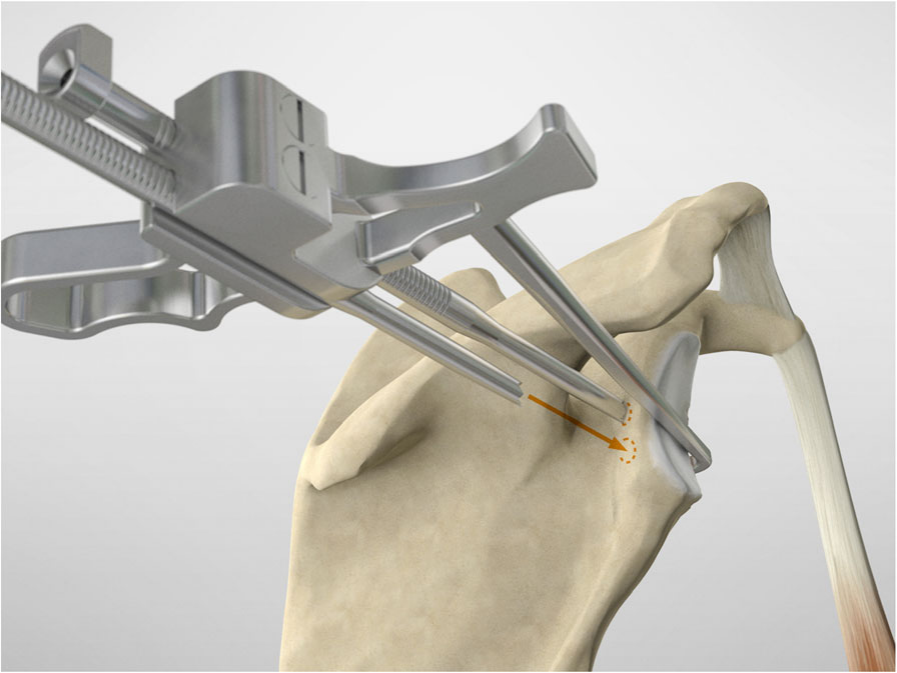
The guide is secured with two bullets placed percutaneously that rest against the posterior glenoid neck

**Fig. 3:**
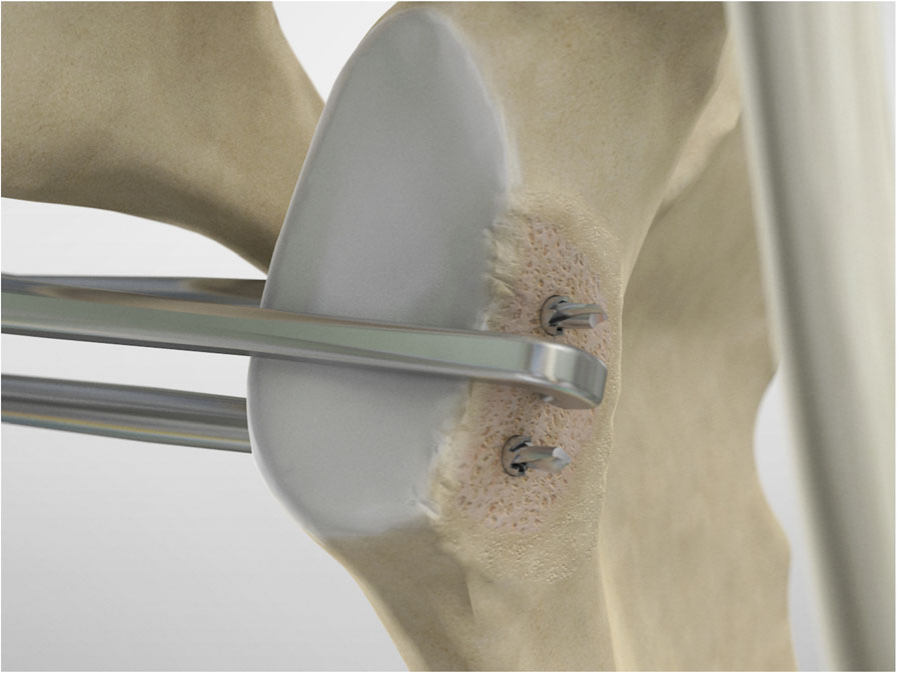
A 2.8 mm sleeved drill is placed through each bullet and advanced under power until exiting from the anterior aspect of the glenoid

**Fig. 4:**
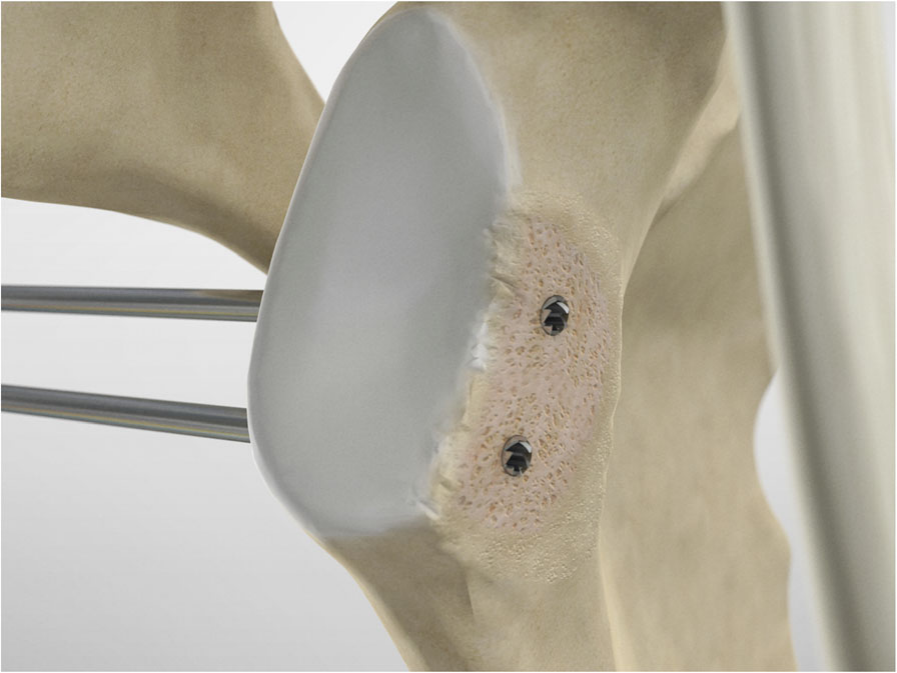
The inner drill is removed, leaving the cannulated outer sleeve in place

The drill guide (Smith & Nephew Inc., USA) is used to ensure that the two drill tunnels are perpendicular to the neck of the glenoid and parallel to each other [27]. It is inserted from a posterior accessory portal and centred on the glenoid defect below the mid-line (Fig. 1). The guide is secured with two bullets placed that rest against the posterior glenoid neck (Fig. 2) percutaneously. To view the jig, the scope is in the anterosuperior portal while the burr is inserted from the mid glenoid portal. A 30° scope is used. Two 2.8 mm sleeved drills are put through each bullet 5 mm under the cortical rim of the glenoid face, parallel to each other and 10 mm separate. The surgeon advances each drill as they appear at the anterior part of the glenoid (Fig. 3), then the internal drill is removed and the cannulated outer sleeve is left in place (Fig. 4).

In this way, an accessible pilot hole through the glenoid is created, and it allows the passage of guidewires for coracoid guidance and final fixation onto the anterior glenoid. The bullets and the guide are removed at this stage, leaving the drill sleeves in place.

To prepare the anterior capsular reconstruction, 3 soft anchors suture-based are placed in the anterior glenoid margin.

**Fig. 5:**
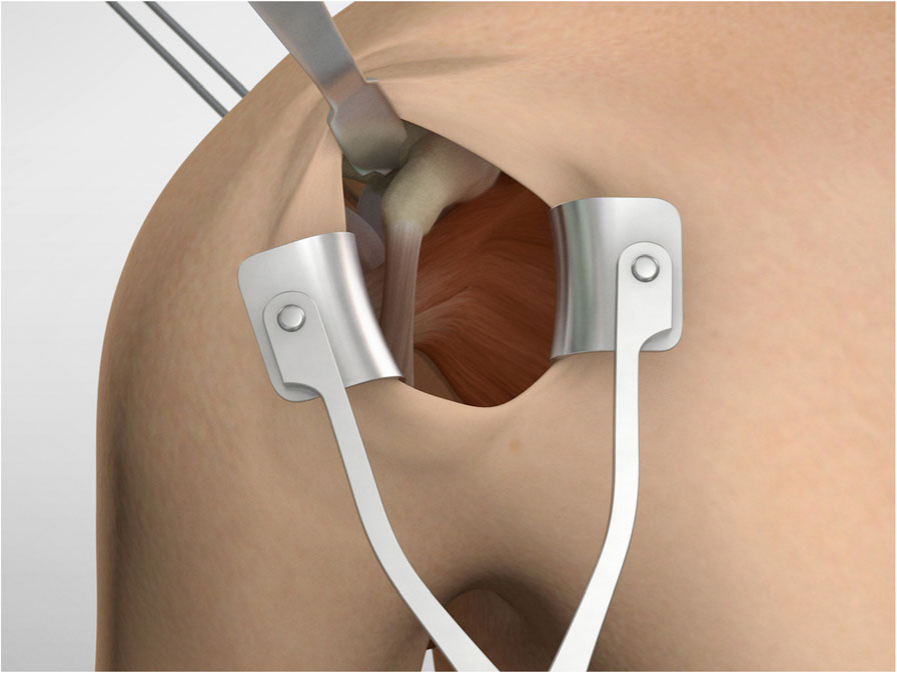
A limited deltopectoral approach, incorporating the mid-glenoid portal at the top of the incision is performed

**Fig. 6:**
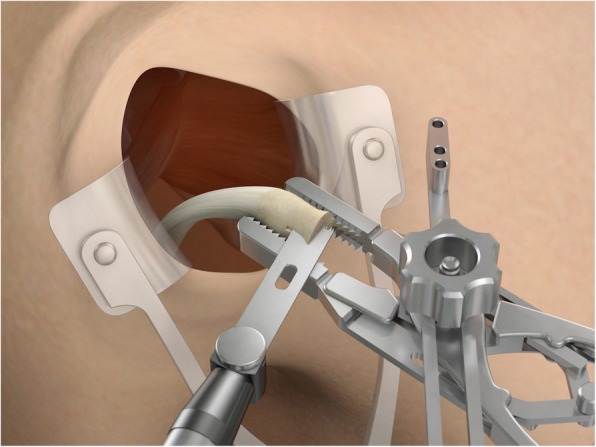
After having performed the coracoid osteotomy, the inferior surface is prepared by removing the soft-tissue and a layer of cortical bone by using the saw

**Fig. 7:**
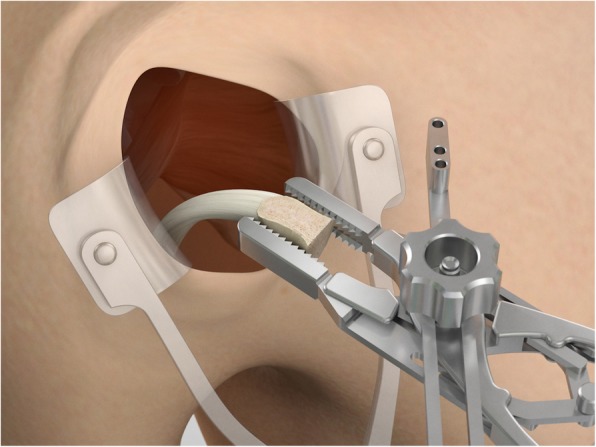
A flat surface of bleeding bone is obtained

**Fig. 8:**
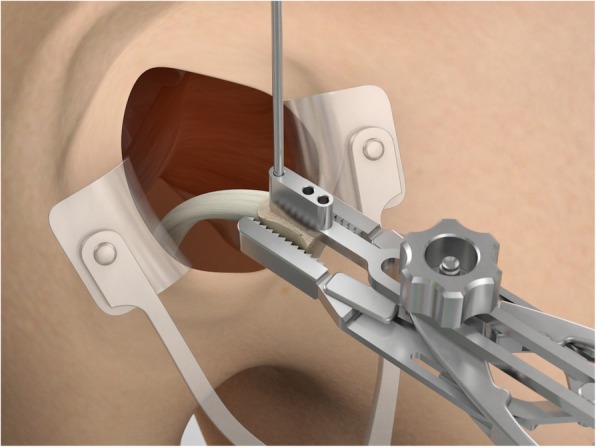
At this point, using the Graft Preparation Tool as a guide, two holes are drilled in the center of the coracoid 10 mm apart and at 5 mm from the lateral edge in order to place the coracoid flush with the glenoid bony margin

**Fig. 9:**
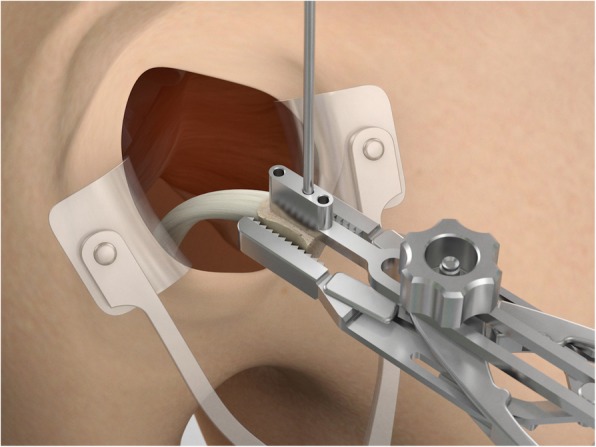
At this point, using the Graft Preparation Tool as a guide, two holes are drilled in the center of the coracoid 10 mm apart and at 5 mm from the lateral edge in order to place the coracoid flush with the glenoid bony margin

**Fig. 10:**
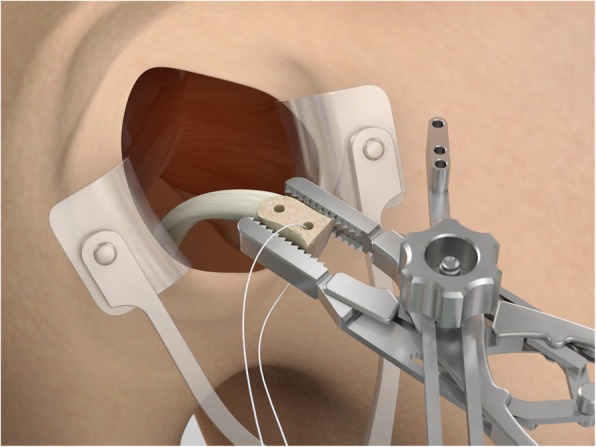
A second tunnel is drilled through the coracoid

**Fig. 11:**
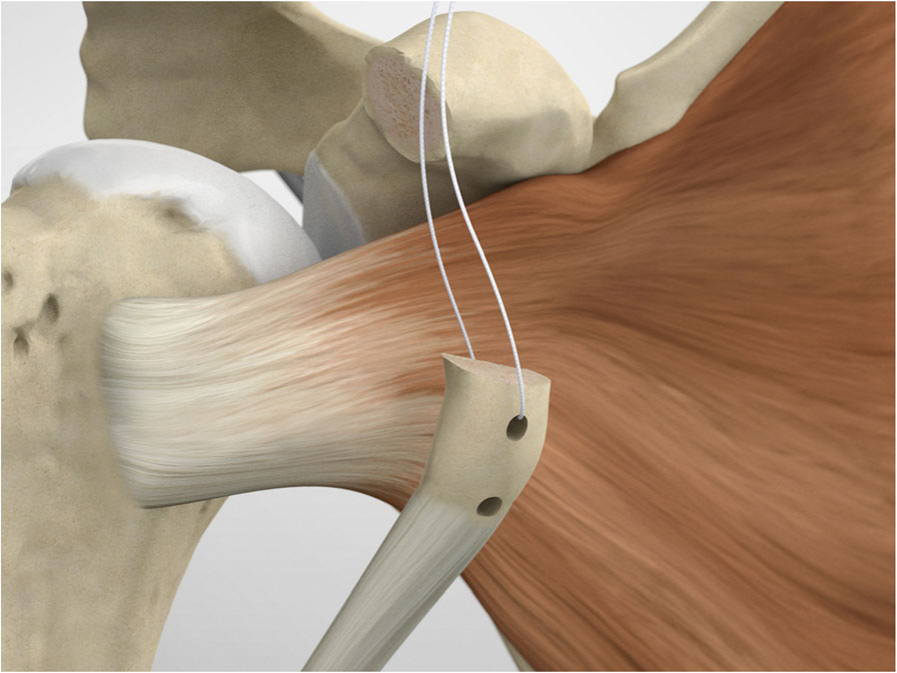
A wire is passed through one hole

The scope and the cannulas are then removed and, employing a restricted deltopectoral access, and the mid-glenoid portal is incorporated on the apex of the cut, as described by Young and Walch [28] (Fig. 5). Following coracoid osteotomy, the inferior surface of the coracoid is prepared by removing the soft-tissue and a layer of cortical bone by using the saw (Fig. 6), to generate a flat surface of bleeding bone (Fig. 7). To place the glenoid bony margin flush with the coracoid, the Graft Preparation Tool is employed as a guide to drill two holes in the centre of the coracoid 10 mm from each other and at 5 mm from the lateral edge (Figs. 8, 9, 10 and 11).

**Fig. 12:**
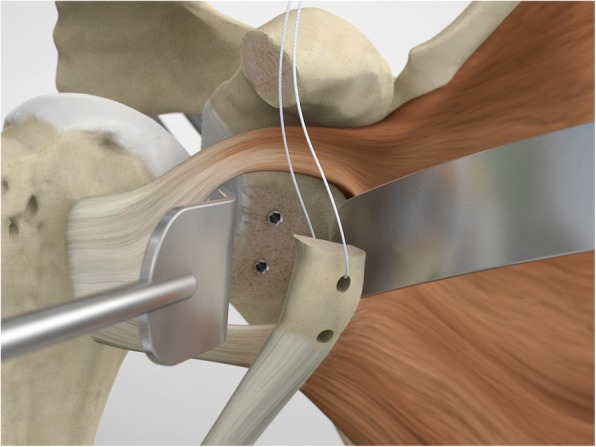
For the glenoid exposure, a subscapularis split is performed at the junction between the superior 2/3 and the inferior 1/3

In the point among the superior 2/3 and the inferior 1/3, the surgeon performs a subscapularis split to expose the glena using a Fukuda retractor and Hohman retractors (Fig. 12).

**Fig. 13:**
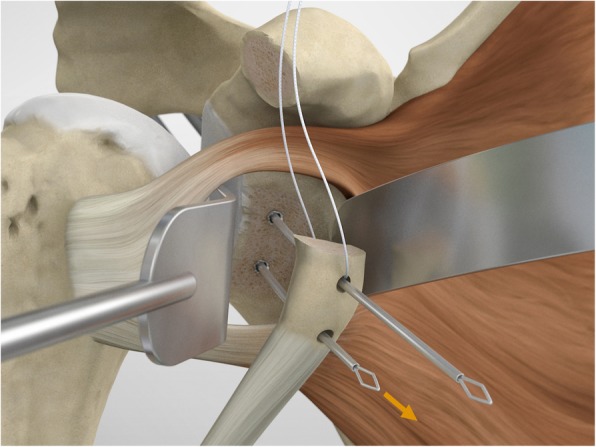
To shuttle and transfer the coracoid process a Suture Retriever is introduced posteriorly into each sleeve, pushed anteriorly and fed through the prepared holes on the coracoid

**Fig. 14:**
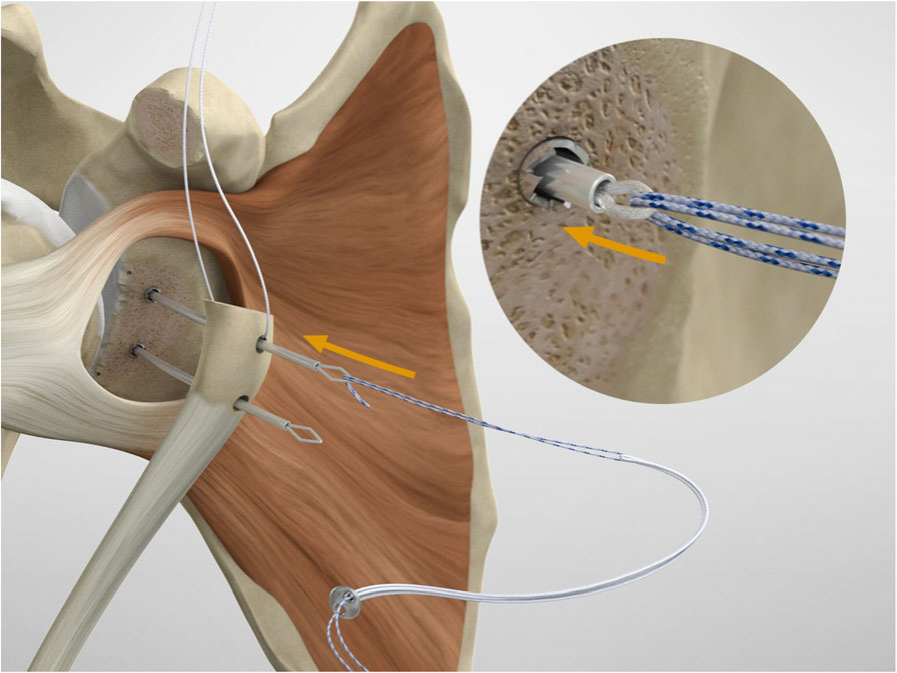
The metal loop on the Suture Retriever is deployed to grab the suture bundle of the anterior peg-style endobutton

**Fig. 15:**
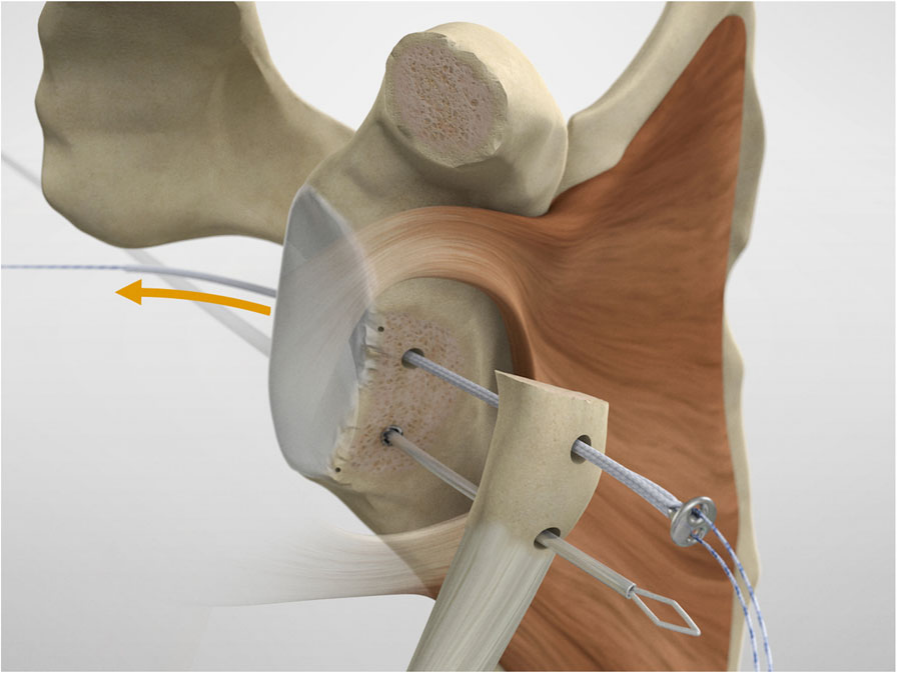
The two drill sleeves are removed prior to pulling the white suture bundle through the glenoid

**Fig. 16:**
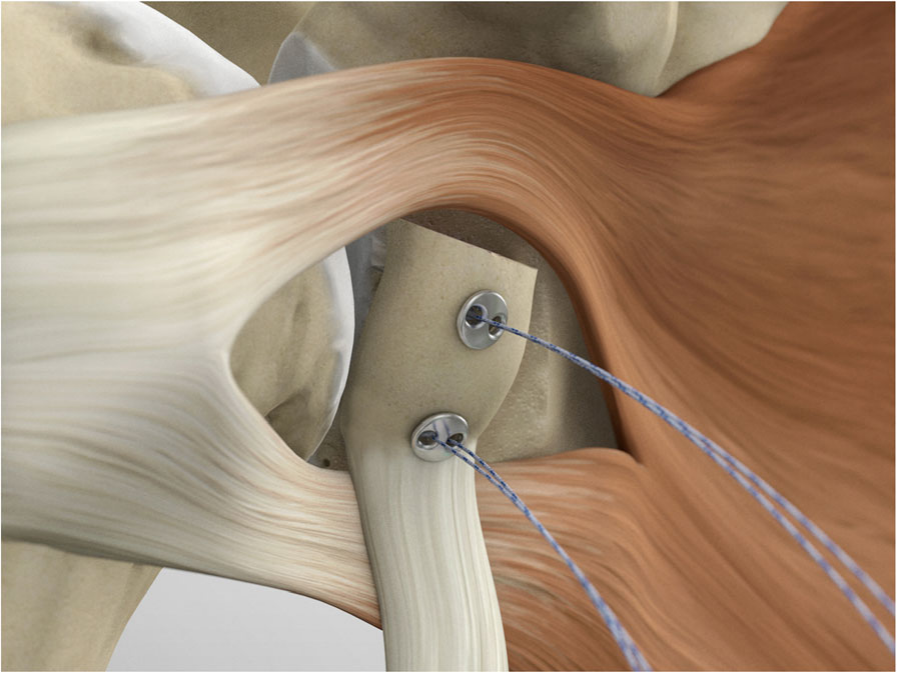
It is important to ensure the endobutton seats itself and lays flush on the surface on the coracoid

To shuttle and transfer the coracoid process, a Suture Retriever is inserted from the posterior side of each sleeve, pushed anteriorly and fed into the prepared holes on the coracoid (Fig. 13). The metal loop on the Suture Retriever is deployed to grab the suture bundle of the anterior peg-style endobutton (Fig. 14). The two drill sleeves are removed before pulling the white suture bundle through the glenoid (Fig. 15). It is essential to ensure the endobutton seats itself and lays flush on the surface on the coracoid (Fig. 16). Coracoid and conjoined tendon are passed through the subscapularis split by pulling on the sutures posteriorly. The coracoid is manipulated until the freshened surface is at level of the anterior surface of the glenoid neck.

**Fig. 17:**
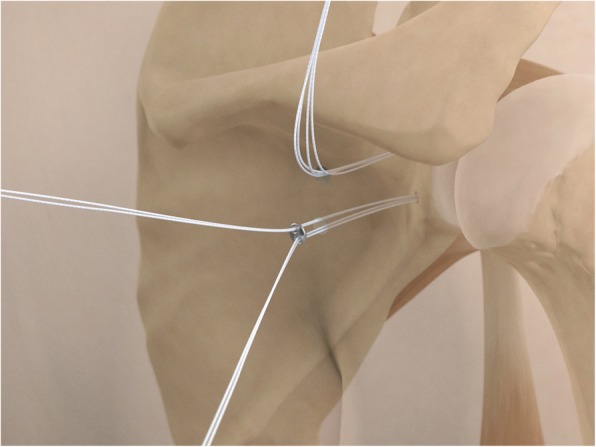
The posterior implants are placed on the implant’s lead suture and are advanced until they sit flush against the posterior face of the glenoid

**Fig. 18:**
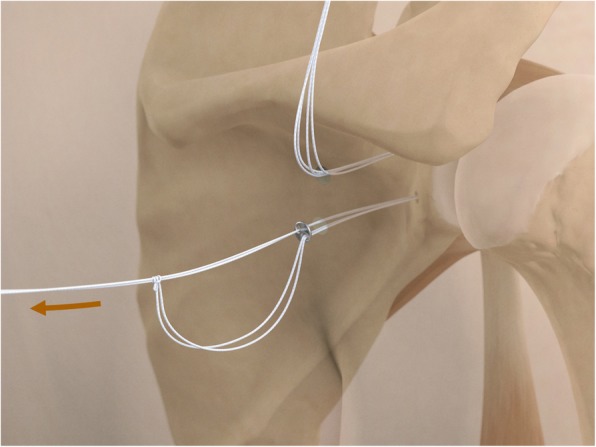
A second endobutton is advanced to the posterior aspect of the glenoid

**Fig. 19:**
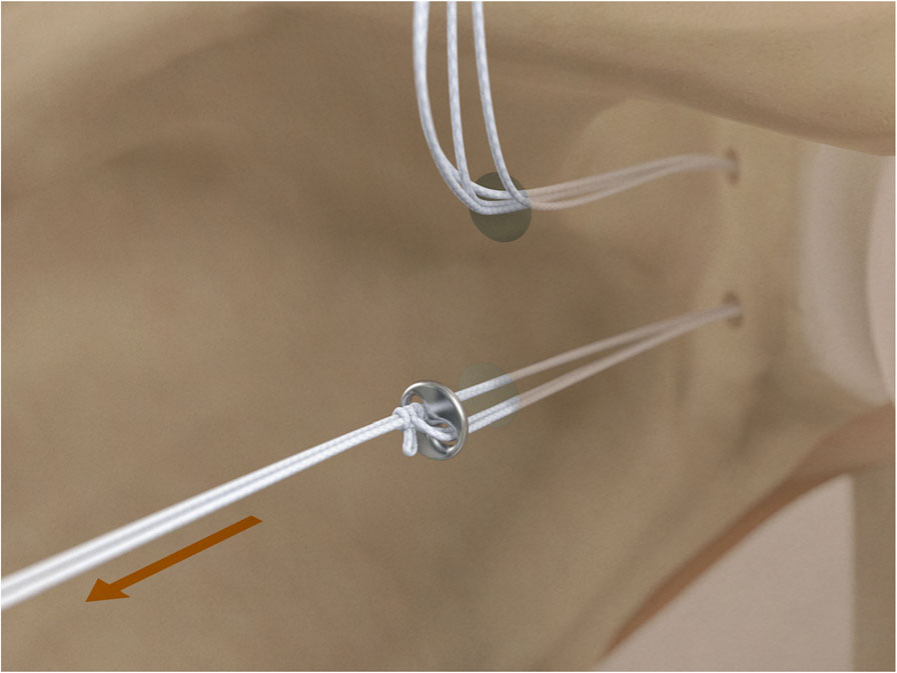
Nice knots are tied

**Fig. 20:**
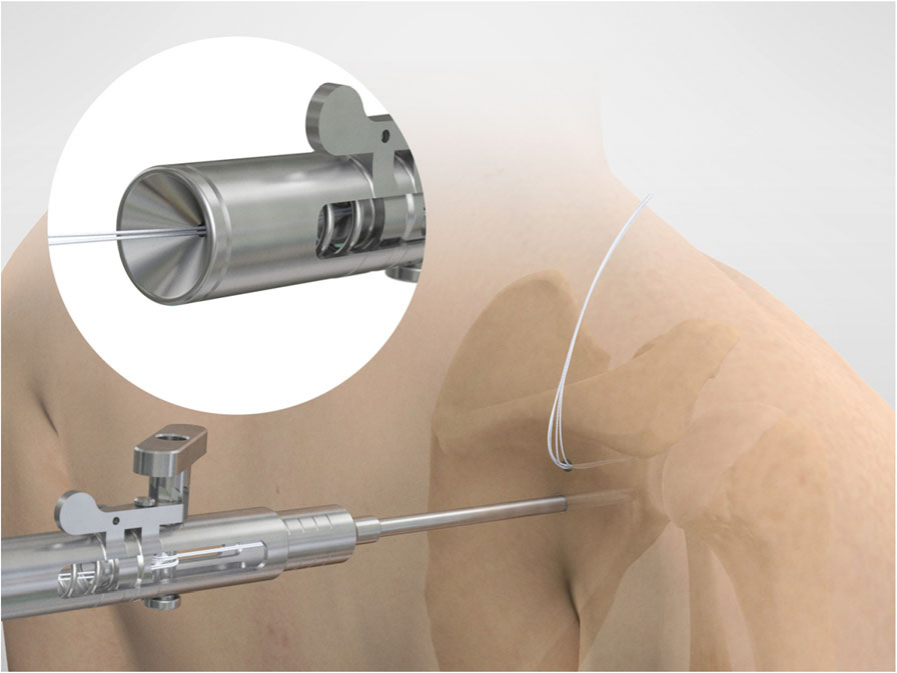
The knot pusher is used to secure the posterior round endobuttons

**Fig. 21:**
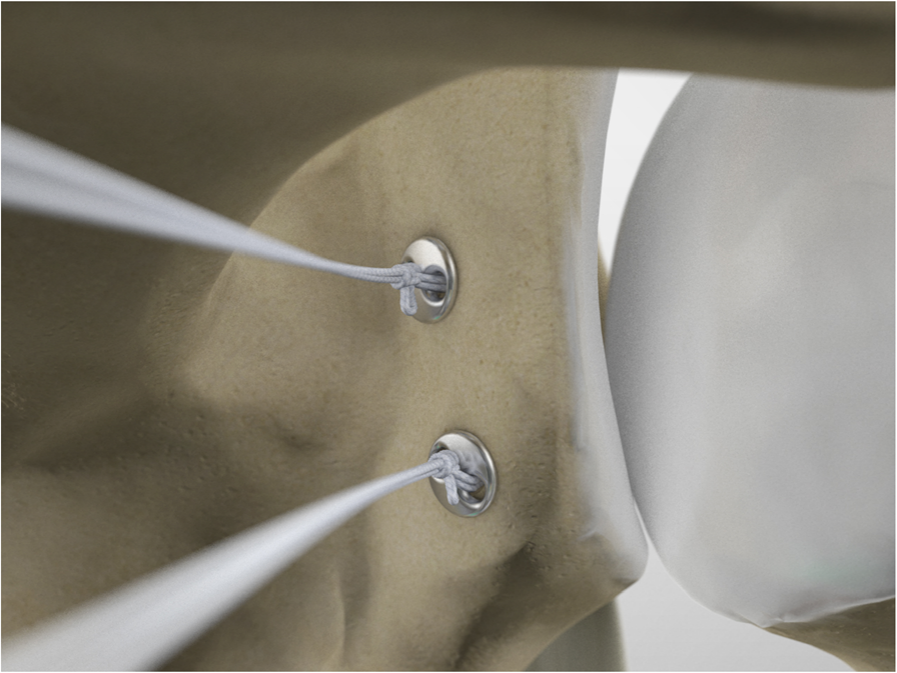
A Nice knot is performed and tensioned with a suture tensioner device to provide strong compression of the graft on the anterior glenoid neck

The posterior implants are advanced to the posterior portion of the glenoid (Figs. 17, 18 and 19), and then the knot pusher is utilized to secure the posterior endobuttons (Fig. 20). A Nice knot is performed and tensioned with a suture tensioner device providing a pressure of the graft on the anterior glenoid neck (Fig. 21). Following the tensioning of the implant, half-stitches are used to secure the posterior knots [29].

**Fig. 22:**
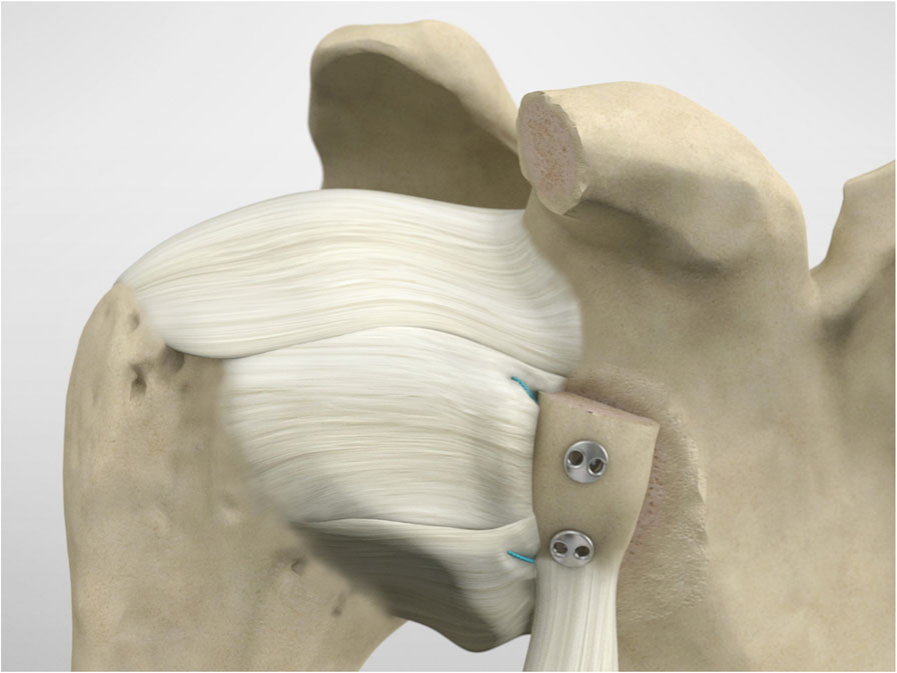
The coracoid and the conjoined tendon remain as extra articular stabilizers

At this point the capulolabral complex is re-inserted on the glenoid side, recovering the suture limbs through the subscapularis split and tightening the knots of the inserted suture-based anchors. The coracoid and the conjoint tendon maintain their extra-articular stabilizer function and position (Fig. 22). Surgical time was on average 89.95 (min 68-max 110, SD 15.45); no intraoperative complications were described.

### Postoperative care

The arm was immobilized with an abduction pillow for the immediate 3 weeks post-surgery. After removal of the sling, passive movements were possible from the 4th week only under supervision. Active movements were allowed from the 4th week but below 90 degrees of flexion and abduction. Complete active movements were permitted after the 7th week. Strengthening exercises were started at the 15th week.

### Clinical evaluation of patients

The following clinical outcomes were evaluated: return to sport at pre-injury level, apprehension and relocation tests positivity, shoulder range of motion, complication or reoperation rate, Rowe score [30], Walch-Duplay score [31] and Subjective Shoulder Value [32]. Subjects satisfaction was assessed, asking whether they would undergo the same procedure if necessary.

At one-year follow-up, each subject was assessed through an *en face* projection of the glenoid on the sagittal plane at CT scan. The surgeon drew a circle above and one below the glenoid flush with its margins using the Osirix software (Pixmeo, Geneva, Switzerland).

The vertical axis of the glenoid is defined as the straight line connecting the central point of both drawn circles, while the horizontal axis as a line crossing the middle of the glenoid height and perpendicular to the vertical axis [33]. The perfect location was identified by the intersection of the line passing for the glenoid equator and the glenoid rim in the horizontal plane [34]. Malpositioning of the bone block was stated if more than 25% of the graft is above the glenoid equator or 1 mm laterally beyond the level of the glenoid rim or 5 mm or more medially to the glenoid rim. However, there is no strict definition of coracoid malposition, but it is diagnosed when the observers consider it too high, medial or lateral.

**Fig. 23:**
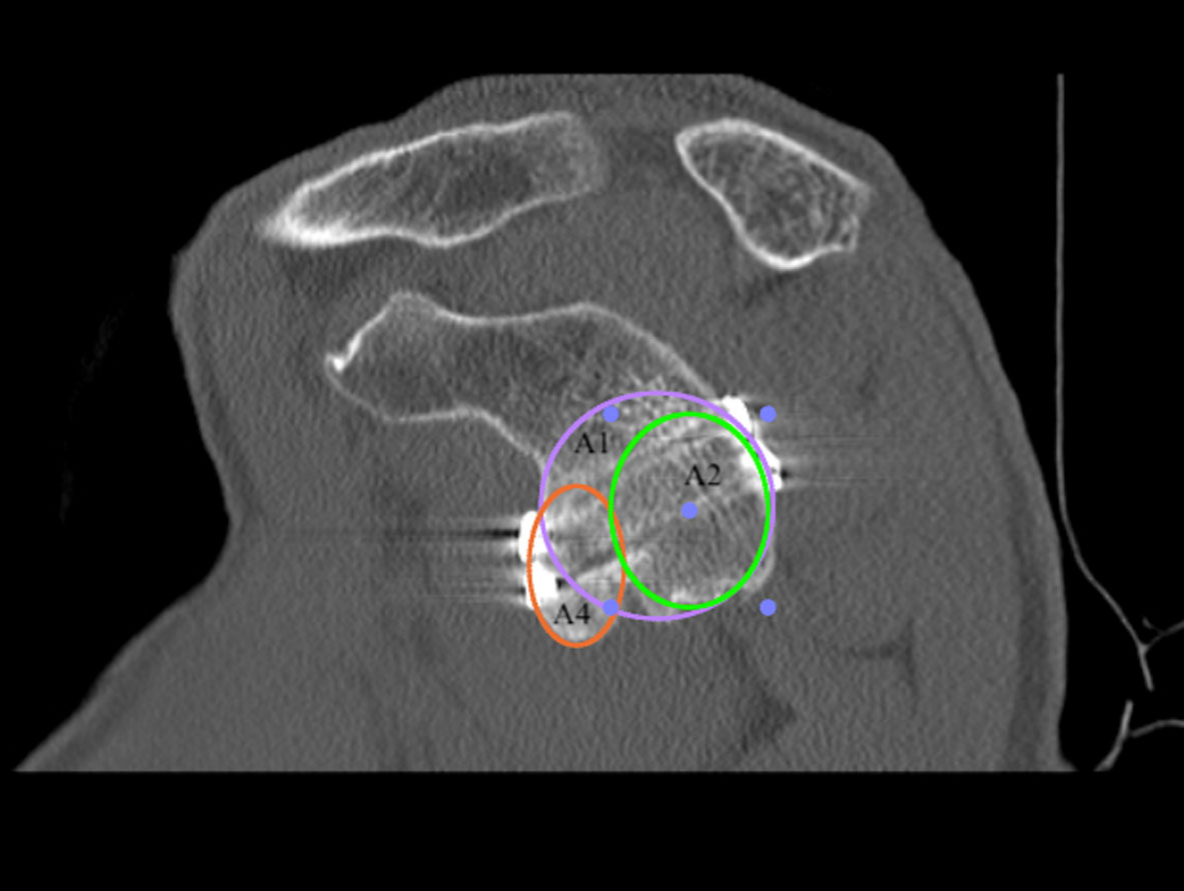
Postoperative CT scan

A graft was considered healed when it was not malpositioned and a bone bridge could be observed between glenoid and graft on the CT. Bony gain was calculated at one-year follow-up on CT scans to evaluate bone remodelling. The original glenoid bone area (A1) and the defective area (A2) were measured to evaluate the bony healing and the effective glenoid area with defect as A3 = A1-A2. Bone graft area (A4) and the A4:A3 ratio, that represents increase of the glenoid surface by the bone block graft, were also measured (Fig. 23) [35].

On the Computer Tomography and the last X-ray control through Grashey and axillary views, the potential migration of the graft, the graft fractures, the possible bone resorption around an endobotton and the osteoarthritis grade according to Samilson and Prieto classification were evaluated by three observers [36]. To avoid potential bias, three different shoulder surgeons not involved in the surgery and also the radiologist, performed all the radiological evaluation.

## Results

The mean follow-up was 32.5 months (range 24–32 months). Sixty patients (44 male, 16 female) were enrolled in this study (mean age 25.5 years, range 18–35 years). The dominant arm was affected in 47 patients (78.3%). No patients were lost to follow-up.

Twenty-eight patients (46.7%) were involved in sports. Seventeen of which participated at a competitive level and eight in high-risk sports (i.e. basketball, rugby, snowboard). Preoperative average ISIS [20] was 4.6 (range 3–6). The deficit of the glenoid was ≥10% on preoperative CT scans (mean 21.5%, 10–33%, SD 5.9) and 45 patients present a Hill-Sachs lesion (75.0%).

At a mean follow-up, 56 of the 60 subjects reported a stable shoulder without postoperative complaints, two (3.3%) had an anterior dislocation after new traumatic injury, and two (3.3%) complained of subjective instability without apprehension and recurrent anterior dislocation or subluxation. At the latest follow-up, four subject complained about painful recurrent anterior instability (Visual analogue scale (VAS 4)) during abduction-external rotation with apprehension. No neurologic complication or infections were recorded.

Most of the patients were satisfied, and 93.3% declared, if needed, that they would undergo the same procedure again.

**Fig. 24:**
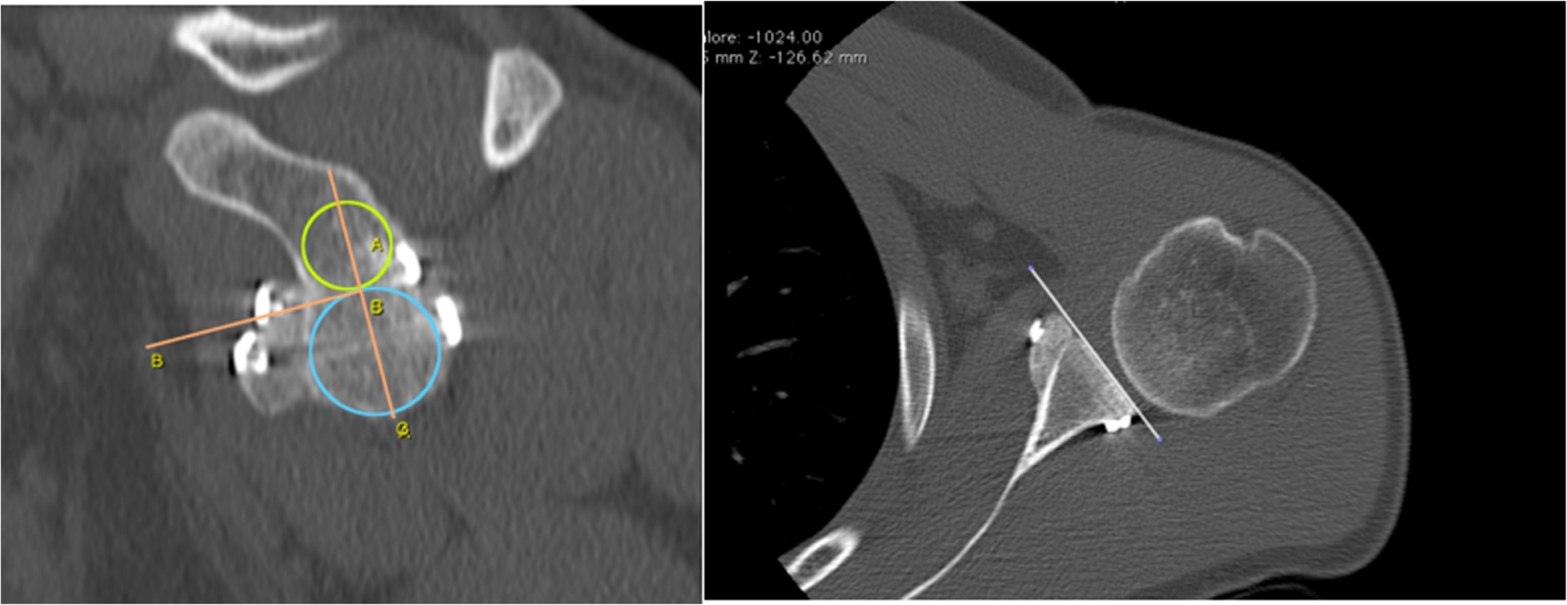
Computed tomography images showing bone graft healing and remodeling after one year

**Fig. 25:**
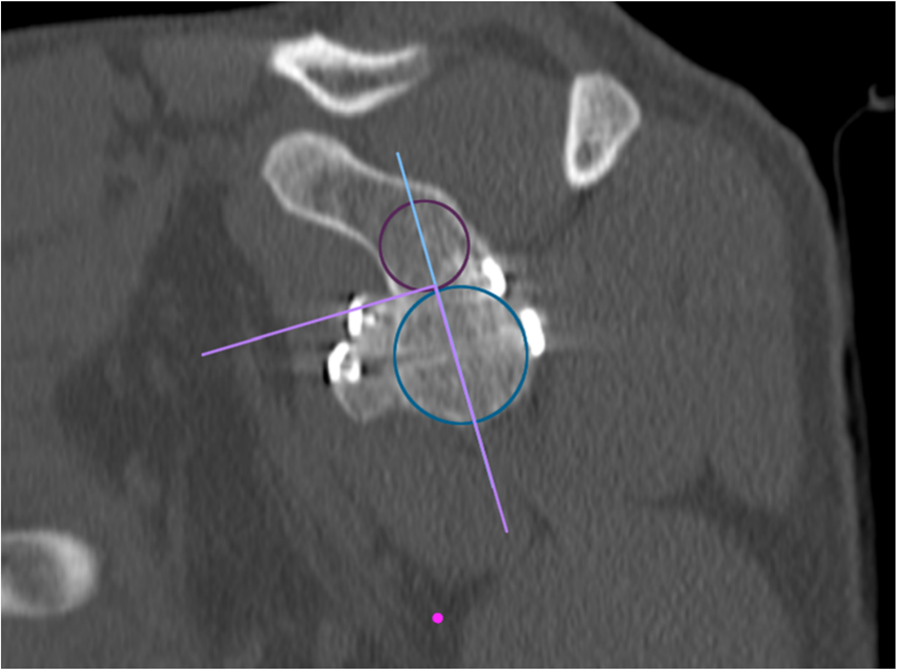
Computed tomography images showing bone graft healing and remodeling after 1 year

Functional and radiological outcomes are summarized in Tables 1 and 2 (Figs. 24 and 25), respectively.

**Table 1:** Functional results

Scoring system	Value
Recurrence Rate	3.3%
Walch-Duplay Score (max 100 points)	92.4 ± 11.6 SD (range 75–100)
Rowe Score (max 100 points)	93.6 ± 12.0 SD (range 75–100)
SSV (max 100%)	88.1 ± 9.4 SD (range 60–100)
Satisfaction grade	94.1% Mostly or Very Satisfied

*SD* Standard Deviation

**Table 2:** Imaging results

Graft positioning	Number of shoulders
Vertical position	
Equator or just under	58 (96.7%)
High	1 (1.7%)
Migrated	1 (1.7%)
Horizontal position	
Flush	59 (98.3%)
Lateral	0
Too medial	0
Migrated	1 (1.7%)

The average loss of external rotation with the elbow at the side was 9.8° (range 0–30°, SD 15.1) and sixteen patients (26.7%) were aware of it.

Twenty-three patients out of 28 came back to sport at the preinjury level (82.1%), 13 out of 17 at a competitive level.

At 1 year follow up, in one of the patients affected by recurrent dislocation, the graft had migrated (1.7%). Of the remnants, one was judged not healed and high positioned (1.7%) and none dissolved.

At one-year follow-up, a glenoid bony gain of 26.3% was recorded (0–37.2%, SD 13.5).

At the latest follow-up, no signs of cartilage joint degeneration were observed in 57 patients (95.0%), and only three patients had grade 1 asymptomatic degenerative changes according to Samilson and Prieto classification [37]. No patients experienced graft fracture; whereas four patients had post-surgical hematoma that resorbed spontaneously. Nerve injuries and infections were not detected.

None of the 60 patients underwent revision surgery.

## Discussion

This arthroscopically-assisted Latarjet technique combines the advantages of an open approach to harvest, prepare and handle the coracoid and the accuracy of an arthroscopically guided graft positioning and fixation. It has the advantage of avoiding dangerous portals medial to the coracoid. The use of an arthroscopic guide to perform the tunnels allows to achieve optimal graft positioning and fixation; the double pair of round-endobuttons avoids rotational instability of the graft.

The site of graft positioning, according to literature, is controversial. Several studies reported the graft placement on the scapular neck, without a specific description of the site [38, 39]; other studies described the site of graft positioning considering the distance from the glenoid rim: the graft could be placed flush with the glenoid rim [40] or medial to the rim [41]. However, the mean rate of redislocation or instability related to graft position is hard to define due to the heterogeneity of data. A systematic review of operative techniques of Bristow-Laterjet procedures reported a mean rate of further redislocation for those techniques that placed the graft at level with the glenoid or medially the glenoid rim of 5.89% (0 to 8.51%) and 0.51% (0 to5.0%) respectively, while the mean rate of instability was 9.17% (0 to 20.7%) and 0.51% (0 to 0.85%), respectively [42].

With the arthroscopically-assisted Latarjet, we found 3.3% of anterior redislocation after new traumatic injury, and 3.3% of subjective instability without apprehension and recurrent anterior dislocation or subluxation.

Nerve injury is a dreadful complication of arthroscopic Latarjet procedure [14]. In this series of 60 patients, no nerve injury was detected. This result could be related to the use of an intraarticular glenoid guide inserted from the posterior portal to avoid an unsafe angulation of the glenoid tunnels. The mini-open approach to harvest, prepare and handle the coracoid with the conjoint tendon is more comfortable, quicker and safer than the arthroscopic procedure avoiding dangerous portals medial to the coracoid.

The round-endobutton fixation and the glenoid guide provides better bony integration of the coracoid than the use of the screws through an anterior approach [27]. Cowling et al. [42] reported an overall mean rate of screws loosening of 1.45% (0 to 4.26%) using two screws for fixation and a mean rate of 2.08% (0 to 6.45%) of loosening using one screw; the mean rate of nonunion was comparable in both groups and revision procedures were 5.16% (0 to 35.7%) using two screws, and in 1.25% using one screw (0.84 to 11.5%) [42]. In our series at 1 year follow up, only one case (1.7%) showed coracoid displacement. Most interestingly, it has been shown in a biomechanical study, that screw fixation and button fixation of coracoid, yield similar fixation strength [43].

The use of the aiming device, with creation of two bony tunnels, permit the realization of two perfectly parallel tunnels with 5mm offset from the glenoid rim [27, 44]. The bony tunnels should be directed parallel to the glena and perpendicular to the graft and glenoid neck. This technique of tunnel’s placement, together with the preparation of the glenoid neck with a flat freshened cancellous bone and the use of a tensioner support give for the fixation, permits to obtain an excellent balanced tension among the two double pairs of round endobuttons [27, 40, 44–46].

The augmented blood flow through the bony tunnels, enhances graft perfusion and therefore probably increases bone integration of the graft [47]. Moreover, the use of round endobuttons reduces the risk of suprascapular nerve lesions and avoids potential complications that seem to occur using screws due to length or direction of the screws, their head impingement with the humeral head or their breakage or loosening [8].

The Bristow-Latarjet technique has several criticisms. The potential loss of external rotation made this procedure unpopolar in some overhead throwing sportsmen [48–50]. However, in the present study, the loss of external rotation with the elbow at side was 9.8° on average, and less than one-third of athletes was aware of it. The great incidence of subjects who return to sport at the preinjury level (82.1%) may be responsible for the restoration of stability and mobility. Moreover, the development of subsequent osteoarthritis is a possible postoperative complication [48, 49]. In this study, 95.0% of subjects had no evidence of gleno-humeral osteoarthritis at last control. However, further results from researches with longer term of follow-up are needed to confirm our preliminary result.

Summarizing, this new mini-open technique allows good positioning of the tunnels and graft preparation.

Weaknesses of this study are the retrospective model and the lack of a control group which precludes definitive conclusions. However, we do intend to perform a prospective randomised trial in future.

Strengths of this study include that patient examination was performed by observers different from the orthopaedic surgeon; a minimal number of subjects was lost at follow-up; every surgical procedure was made by the same senior surgeon; CT scans were performed postoperatively.

## Conclusions

This technique of arthroscopically assisted Latarjet combines mini-open and arthroscopic approach to improve the precision of the bony tunnels in the glenoid and coracoid placement, minimizing any potential risk of neurologic complications. It can be an option in subjects with anterior gleno-humeral instability and glenoid bone defect. Further studies should be performed to confirm our preliminary results.

## Data Availability

The datasets used and/or analyzed during the current study available from the corresponding author on reasonable request.

## Abbreviations

CT: Computer tomography
ISIS: Instability Severity Index Score
VAS: Visual analogue scale
